# Spontaneous Nystagmus in the Dark in an Infantile Nystagmus Patient May Represent Negative Optokinetic Afternystagmus

**DOI:** 10.3389/fneur.2018.00151

**Published:** 2018-03-14

**Authors:** Ting-Feng Lin, Christina Gerth-Kahlert, James V. M. Hanson, Dominik Straumann, Melody Ying-Yu Huang

**Affiliations:** ^1^Department of Neurology, University Hospital Zurich, University of Zurich, Zurich, Switzerland; ^2^Neuroscience Center Zurich (ZNZ), University of Zurich and ETH Zurich, Zurich, Switzerland; ^3^Department of Ophthalmology, University Hospital Zurich, Zurich, Switzerland; ^4^Neuroimmunology and Multiple Sclerosis Research, Clinic for Neurology, University Hospital Zurich, University of Zurich, Zurich, Switzerland

**Keywords:** infantile nystagmus syndrome, optokinetic response, optokinetic afternystagmus, smooth pursuit, smooth pursuit afternystagmus, albinism

## Abstract

Abnormal projection of the optic nerves to the wrong cerebral hemisphere transforms the optokinetic system from its usual negative feedback loop to a positive feedback loop with characteristic ocular motor instabilities including directional reversal of the optokinetic nystagmus (OKN) and spontaneous nystagmus, which are common features of infantile nystagmus syndrome (INS). Visual input plays a critical role in INS linked to an underlying optic nerve misprojection such as that often seen in albinism. However, spontaneous nystagmus often continues in darkness, making the visual, sensory-driven etiology questionable. We propose that sensorimotor adaptation during the constant nystagmus of patients in the light could account for continuing nystagmus in the dark. The OKN is a stereotyped reflexive eye movement in response to motion in the surround and serves to stabilize the visual image on the retina, allowing high resolution vision. Robust negative optokinetic afternystagmus (negative OKAN), referring to the continuous nystagmus in the dark with opposite beating direction of the preceding OKN, has been identified in various non-foveated animals. In humans, a robust afternystagmus in the same direction as previous smooth-pursuit movements (the eye’s continuous tracking and foveation of a moving target) induced by visual stimuli has been known to commonly mask negative OKAN. Some INS patients are often associated with ocular hypopigmentation, foveal hypoplasia, and compromised smooth pursuit. We identified an INS case with negative OKAN in the dark, in contrast to the positive afternystagmus in healthy subjects. We hypothesize that spontaneous nystagmus in the dark in INS patients may be attributable to sensory adaptation in the optokinetic system after a sustained period of spontaneous nystagmus with directional visual input in light.

## Introduction

Infantile nystagmus syndrome (INS), also known as congenital nystagmus, is an ocular motor disorder which is commonly identified in infants less than 2–3 months old (1). INS patients usually exhibit involuntary horizontal eye movements (1). Genetic sequences have suggested that a variety of gene mutations lead to the disruption of neurophysiological functions in afferent visual pathways, ocular motor system, and the mechanisms involved with extraocular muscle innervations (2). Different disease mechanisms and models have been proposed for INS: Yonehara et al. reported that the FRMD7 gene mutation caused a significantly reduced asymmetric inhibition of starburst amacrine cells to direction-selective ganglion cells in the retina (3). Huang and colleagues suggested that a positive feedback optokinetic controlling system underlying optic nerve fiber misrouting can lead to INS-like ocular motor behaviors in animal models and in humans (4–7). Earlier, Optican and Zee also proposed a positive feedback loop model which results in an unstable neural integrator (8). Jacobs and Dell’Osso developed a model based on an underdamped smooth-pursuit system (9, 10). Further, Brodsky and Dell’Osso proposed that malfunction of the smooth-pursuit system would cause an uncontrolled optokinetic system (11). Harris and Berry suggested that abnormal eye oscillations may develop due to a poor, high spatial frequency, contrast sensitivity (12, 13). Akman et al. used a nonlinear dynamics model based on an abnormal saccadic system to predict nystagmus (14). Berg et al. reported changes to extraocular muscle properties in INS patients, which suggested an adaptation mechanism at the effector level due to deficient motor innervations (15). Even though all of these models were proposed to help explain the pathological mechanism underlying INS, to date no consensus exists as to which is most credible (2).

Among the known genetic mutations, there is a group of patients who share a common pathological phenotype, ocular hypopigmentation, which is caused by the reduction of melanogenesis. Oculocutaneous albinism (OCA) is an autosomal-recessive disorder in which pigmentation of the hair, skin, and eyes is reduced (16). Ocular albinism (OA) is an X-linked disorder, which shows hypopigmentation only in the eyes (17, 18). All types of OCA and OA patients have been reported to exhibit INS (17–25). Huang et al. demonstrated that the zebrafish mutant *belladonna* (*bel*), which exhibit abnormal retinal ganglion cell (RGC) projections (a defect also commonly found in albino patients), exhibited reversed optokinetic nystagmus (OKN) and spontaneous nystagmus, both of which are often seen in INS patients (4, 5). In a subsequent publication, the same authors demonstrated that the nystagmic eye movements found in *bel* qualitatively resemble those seen in INS patients (26). It was proposed that the reversed OKN and spontaneous nystagmus were caused by the underlying abnormal RGC projection causing a transformation of the optokinetic system from a negative feedback loop to a positive feedback loop (4, 6). With normal negative feedback control, the retinal slip velocity is used as the error signal, which drives the eyes to move with the moving surround in order to reduce the retinal slip; in contrast, the motor output (i.e., eye movement) of a positive feedback loop would further increase the error signal (i.e., retinal slip velocity). However, while data from INS models (4–7) supporting the abnormal pathway hypothesis of INS can be taken as evidence for the causal role of afferent visual deficits, one remaining challenge is elucidating how the primary sensory input contributes to the pathological mechanism of INS without visual input, since patients also show nystagmus in the dark (27). Shawkat reported the spontaneous reversal of nystagmus beating direction in the dark in manifest latent nystagmus (MLN) and INS patients (28). While the author proposed the non-seeing eye in these patients as the potentially dominant eye and adapted the MLN mechanism to explain the nystagmus in the dark, the evident reversal of nystagmus beating directions from light to dark could actually be attributed to a visual sensory adaptation during the nystagmus in the light.

Both smooth-pursuit and optokinetic ocular motor subsystems have been suggested to contribute to the pathological eye movements in INS (2, 11). Smooth pursuit (or foveal pursuit) refers to the voluntary tracking of moving objects *via* cortical pursuit pathways (11, 27). During the foveal smooth pursuit, it is necessary for the visual target to be located in the visual field of the fovea or, in the case of perifoveal smooth pursuit, perifovea so that the eyes can lock onto the target (27); in other words, a functional fovea is essential in order to perform smooth pursuit. Continued smooth-pursuit behavior was reported to induce an afternystagmus in darkness in the same beating direction for at least 3 min (29). Afoveation is commonly found in INS patients, of whom many are affected with albinism (2). Thus, it is conceivable that smooth-pursuit function may be compromised in INS due to afoveation as well as the nystagmic eye movements. The subcortical optokinetic pathways are responsible for the OKN, which is an involuntary tracking of a moving surround or a large field of motion in the surround (11, 30). Positive optokinetic afternystagmus (OKAN) describes a short-lived (<1 min) persisting eye movement in darkness after the cessation of optokinetic stimulation (31–33). Besides positive OKAN, a reversed afternystagmus (i.e., associated with beating in the opposite direction) of longer duration has been reported in different species including human adults (32), infants (34), monkeys (35, 36), rabbits (37–39), cats (40, 41), and rats (42). This condition is also known as negative OKAN or, in some cases, secondary OKAN or reversed post-optokinetic nystagmus. However, the underlying mechanisms relating to this phenomenon remain unknown and existing studies show wide variability. In general, the presence of negative OKAN depends on the duration of the optokinetic stimulation. Animal studies have shown that a longer period of stimulation leads to a shorter positive OKAN followed by a longer negative OKAN (36, 39).

Abnormal binocular vision (i.e., monocular occlusion or strabismus) has been reported to result in impaired smooth-pursuit function, which may further lead to lack of the normal cortical suppression of the optokinetic pathways by the smooth-pursuit system (11, 28, 43, 44). Based on clinical observations of the spontaneous reversal of nystagmus beating direction in darkness in MLN patients with single healthy eyes or an INS patient with convergent strabismus (28), and the robust negative OKAN observed in animals lacking evident smooth-pursuit functions (37, 38, 42), we propose a new hypothesis of the pathological mechanism underlying INS in darkness. The nystagmus in darkness can develop *via* an adaptive process in the optokinetic system during a sustained period of spontaneous nystagmus in the light. In our present study, we recorded a clear negative OKAN in an INS patient with iris transillumination and foveal hypoplasia. In a healthy subject examined using the same experimental paradigm, we observed an aftereffect of eye movements predominantly in the same direction of the preceding stimulus, which we believed to be afternystagmus following smooth pursuit. Until now, there has been no plausible explanation for the occurrence of the spontaneous nystagmus in darkness following the visual input-related nystagmus in the light, which brings into question the role of aberrant visual sensory processing in INS etiology (4, 6). Based on the outcome of our current study, we hypothesize that nystagmus in the dark in INS patients may be a result of sensorimotor adaptation in the optokinetic system, *via* a similar adaptive process to that observed commonly in afoveated animals and manifested as negative OKAN.

## Materials and Methods

### Medical Information of the Participants

**Figure 1:**
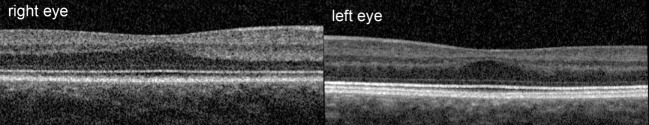
Optical coherence tomography scan showed the foveal hypoplasia.

**Figure 2:**
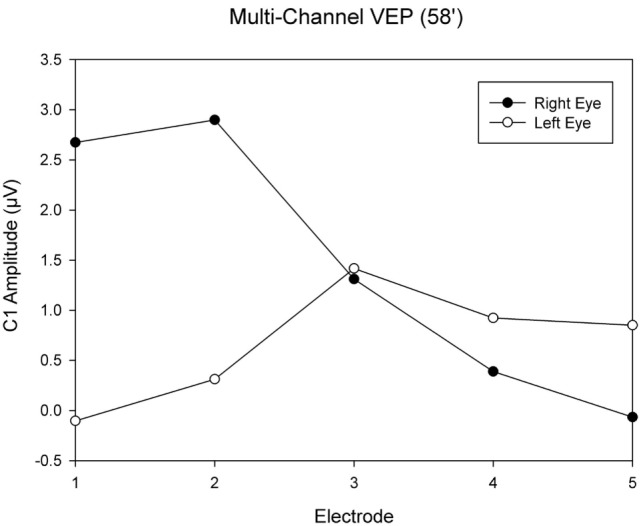
Visual evoked potential (VEP) topography revealed asymmetric response localization over the two cerebral hemispheres in the left and right eye pattern onset responses.

This was an observational study on a 19-year-old female INS patient with mild OCA. The diagnosis was based on the results of the clinical examination; no genetic analysis was performed. There was no family history of OCA or OA. Visual acuity with her myopic astigmatism corrected was 20/50 and 20/40 in the right and left eye, respectively. Ophthalmological examination revealed iris transillumination, chorioretinal hypopigmentation, and macular hypoplasia, but no optic nerve hypoplasia. Foveal hypoplasia was defined as grade 2 to 3 in both eyes (45) by optical coherence tomography (OCT) (Figure 1). Analysis of multi-channel pattern-appearance visual evoked potentials (VEP) revealed asymmetric response localization over the two cerebral hemispheres consistent with previously described findings in albinism (Figure 2) (46).

The healthy subject was a 29-year-old male, who had no ocular or ocular motor abnormalities. Both subjects described herein gave their informed consent for inclusion in this report.

### Experimental Apparatus

Subjects sat upright on a fixed chair surrounded by a custom-built optokinetic drum, which was constructed by a horizontally rotatable cylinder (radius: 74 cm) painted with black and white vertical stripes of width: 9.69 cm (spatial frequency 0.067 cycles/degree). The rotation of the optokinetic drum was driven by a servo-controlled motor-driven axes turntable system (Acutrol^®^ ACT2000, Acutronic, Switzerland Ltd.). A remotely controlled light source was mounted on the ceiling of the cylinder. During the recording, the subject was restrained by safety belts around the feet and trunk with the head being stabilized by a headrest.

### Recording of Eye Movements

Horizontal eye movements were recorded using a head-mounted monocular video-oculography (VOG) device (EyeSeeCam), running at 220 Hz (47, 48), employing an infrared light source and an infrared sensitive camera. Pupil positions were detected by the camera and analyzed by the VOG system online. Eye positions were calibrated before each recording and the data were analyzed offline by custom-built software written in MATLAB (Mathworks, Natick, MA, USA), version (R2014a).

### Experimental Procedure

Before the experiment, the INS patient was first tested for directional bias of eye beating in each eye. The patient sat inside the optokinetic drum and was instructed to look at the stationary vertical stripes for 5 min. During the monocular testing, only the viewing eye was recorded. After both eyes were tested, the left eye was chosen for the subsequent optokinetic test as it showed less directional bias during its spontaneous nystagmus. The control subject showed no clear eye dominance and, therefore, the left eye was also chosen for the test.

During the experiment, the left eye position was recorded while the right eye was covered by soft tissues. The subject was recorded in complete darkness for 1 min followed by a 10-min optokinetic stimulation with a constant stimulus velocity of 30°/s in the clockwise direction. Subsequently, the light was switched off for another minute before the stimulus changed to the counterclockwise direction for another 10-min period. The experiment was concluded with another 1-min recording in total darkness. During the optokinetic stimulation, subjects were instructed to follow the horizontally moving vertical stripes. Left eye movements were recorded throughout the entire experimental procedure.

## Results

**Figure 3:**
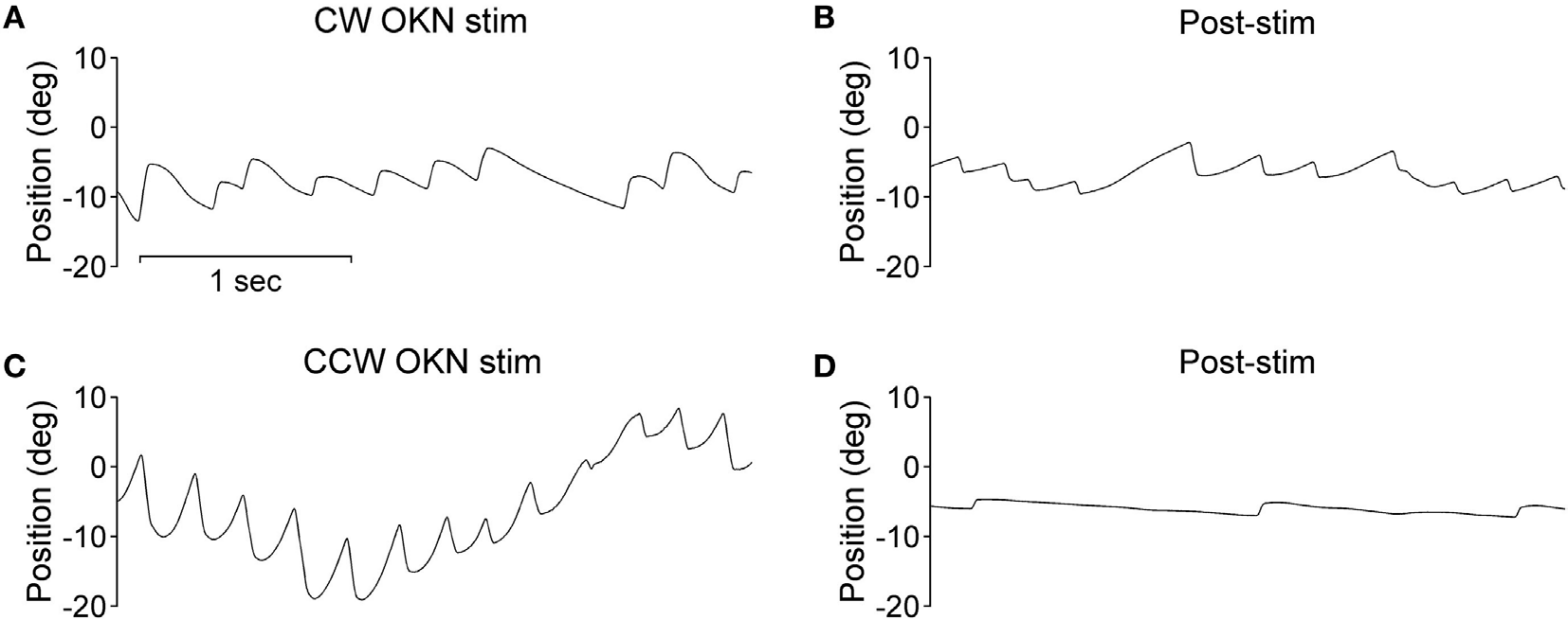
Optokinetic nystagmus (OKN) and afternystagmus of the infantile nystagmus syndrome patient. Plots on the left demonstrate eye position traces during optokinetic stimulation in **(A)** CW (clockwise) and **(C)** CCW (counterclockwise) directions. Plots on the right demonstrate eye position traces in darkness subsequent to the 10-min optokinetic stimulations in **(B)** CW (clockwise) and **(D)** CCW (counterclockwise) directions. OKN stim, optokinetic stimulation phase; post-stim, post-stimulation phase.

**Figure 4:**
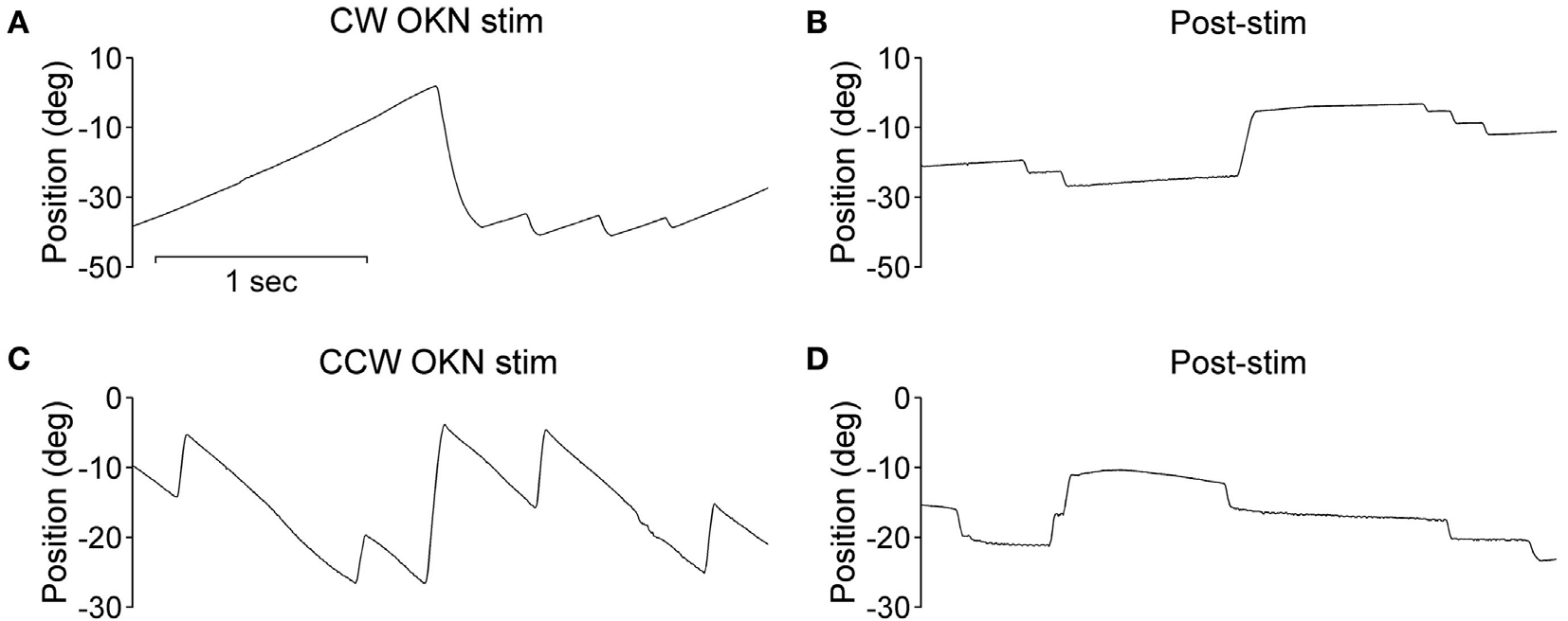
Optokinetic nystagmus (OKN) and afternystagmus of the healthy subject. Plots on the left demonstrate eye position traces during optokinetic stimulation in **(A)** CW (clockwise) and **(C)** CCW (counterclockwise) directions. Plots on the right demonstrate eye position traces in darkness subsequent to the 10-min optokinetic stimulations in **(B)** CW (clockwise) and **(D)** CCW (counterclockwise) directions. OKN stim, optokinetic stimulation phase; post-stim, post-stimulation phase.

During the optokinetic stimulation with a constant stimulus velocity of 30°/s in both clockwise and counterclockwise directions, the INS patient showed a reversed optokinetic eye reflex (Figures 3A,C); by contrast, the healthy subject displayed a typical optokinetic eye reflex with the slow tracking eye movement in the same direction of the drum rotation (Figures 4A,C). After the clockwise optokinetic stimulation, the eye movements of the healthy subject continued in the same direction, with reduced velocity, in the subsequent complete darkness (Figure 4B); in contrast to this, eye movements of the INS patient reversed, with the eye beating in the opposite direction (Figure 3B). In the complete darkness following counterclockwise optokinetic stimulation, the eye movements of the healthy subject continued with reduced velocity mainly in the same direction, but with occasional isolated reversed beatings (Figure 4D). In the case of the INS patient, interestingly, eye movements continued in the same beating direction in complete darkness during the first 50 s and then reversed to the opposite direction (Figure 3D). However, under binocular viewing conditions, both the forward afternystagmus in the healthy and the reversed afternystagmus in the INS patient were much more pronounced (data not shown).

**Figure 5:**
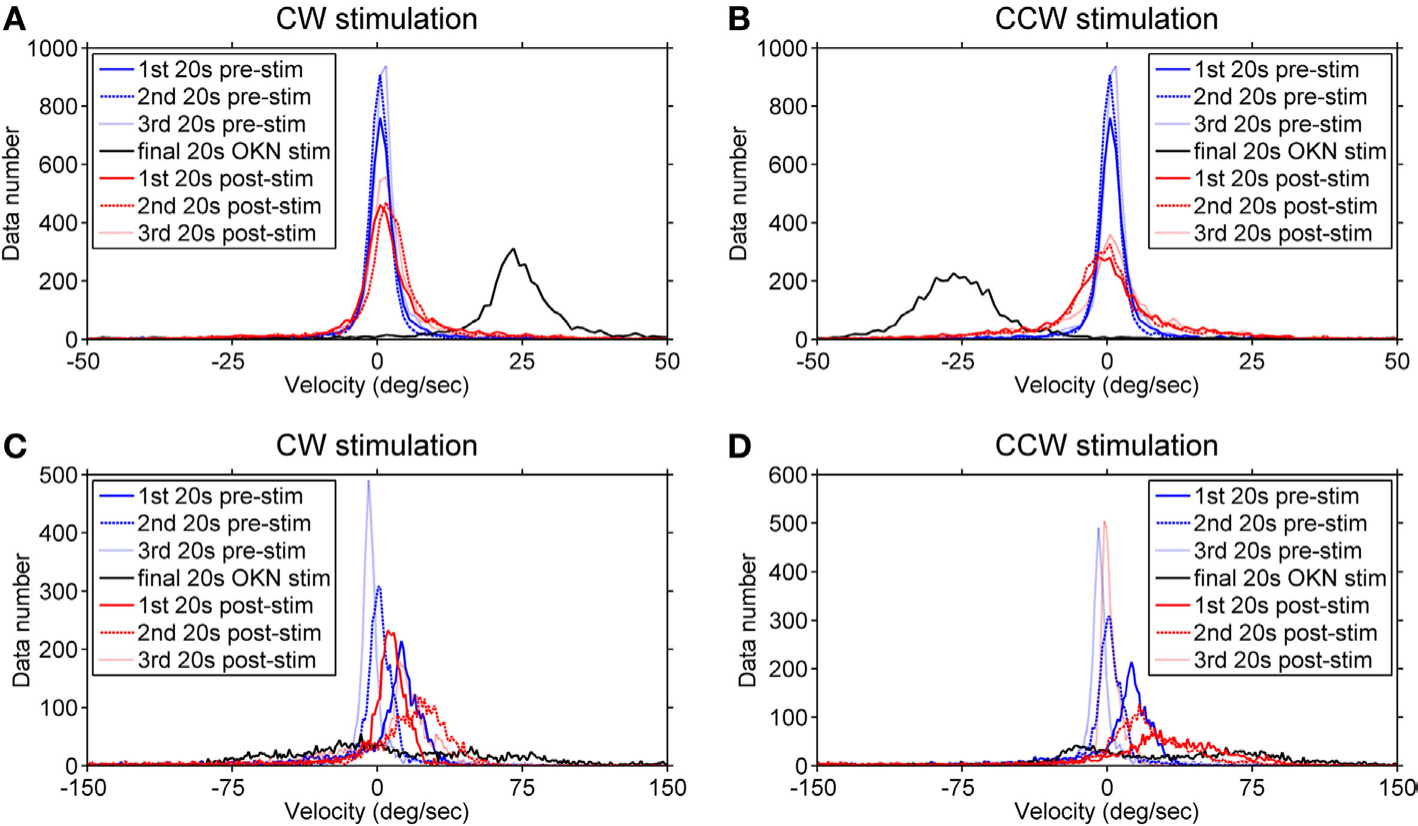
Velocity distribution before, during, and after the optokinetic stimulation. The data numbers within each time window of 20 s were plotted against velocity ranks (every 1°/s). Plots on the top demonstrate the velocity distribution of the healthy subject **(A,B)** and, at the bottom, of the infantile nystagmus syndrome (INS) patient **(C,D)**. The velocity distributions during the pre-stimulation phase are shown in **(A,B)** of the healthy subject and **(C,D)** of the INS patient. Plots on the left demonstrate the velocity distribution during and after the clockwise (positive) stimulation while the plots on the right demonstrate the velocity distribution during and after the counterclockwise (negative) stimulation. The velocity distributions during the pre-stimulation phase are shown as blue line (first 20 s), blue dashed line (second 20 s), and blue dotted line (third 20 s); during the final 20-s stimulation phase as black line; during the post-stimulation phases as red line (first 20 s), red dashed line (second 20 s), and red dotted line (third 20 s). 1st 20 s pre-stim = first 20 s of the pre-stimulation phase; 2nd 20 s pre-stim = second 20 s of the pre-stimulation phase; 3rd 20 s pre-stim = third 20 s of the pre-stimulation phase; final 20 s optokinetic nystagmus (OKN) stim = final 20 s of the optokinetic stimulation phase; 1st 20 s post-stim = first 20 s of the post-stimulation phase; 2nd 20 s post-stim = second 20 s of the post-stimulation phase; 3rd 20 s post-stim = third 20 s of the post-stimulation phase.

To better visualize the directional relationship of the eye velocities over time under different visual conditions, we computed the velocity distribution of the eye movements in each viewing condition within time windows of 20 s. Data numbers were plotted versus velocity ranks (every 1°/s) and we compared the three 20-s time windows of the pre-stimulation dark phase (eye movements in darkness before the stimulation), the final 20 s of the optokinetic stimulation phases, and the three 20-s time windows of the post-stimulation dark phases (eye movements in darkness after optokinetic stimulation) (Figure 5). The healthy subject’s eye velocity distribution peaks fell tightly around 0°/s for all three 20-s periods of the pre-stimulation dark phase (Figures 5A,B); in comparison, the INS patient showed a broader velocity distribution, as well as a directional bias toward the positive velocity (i.e., clockwise direction) during the pre-stimulation dark phase (Figures 5C,D). However, during this 1-min dark period the eye velocity markedly reduced over time with the distribution peak shifting toward 0°/s (Figures 5C,D).

During the final 20 s of the optokinetic stimulation phases in both directions, the healthy subject showed clear velocity distributions consistent with the stimulus directions (Figures 5A,B); however, the INS patient showed a broad velocity distribution with more data falling over velocities in the opposite direction of the stimulus (Figures 5C,D). After the clockwise optokinetic stimulation, the healthy subject showed a clear data distribution over velocities in the same direction as during the stimulation phase (Figure 5A); for the post counterclockwise optokinetic stimulation phase, the healthy subject again showed a data distribution over velocities in the same direction as during the stimulation phase (Figure 5B). Moreover, the velocity distributions of both post-stimulation dark phases were broader than in the pre-stimulation dark phase (Figures 5A,B). After the clockwise optokinetic stimulation, the INS patient showed a data distribution over velocities in the opposite direction compared to the stimulation phase (Figure 5C). However, after the counterclockwise optokinetic stimulation, the patient showed a data distribution first over velocities in the same direction as during the stimulation phase, but then eye velocities markedly reduced over time (Figure 5D).

## Discussion

### Comparison of Nystagmus in Darkness After Prolonged Smooth Pursuit and Optokinetic Tracking

Previous studies have reported that background movements of the whole visual field while the eyes remained fixed on a stationary central target led to afternystagmus in darkness with slow phase eye movements in the opposite direction of the preceding background movement (49, 50). This suggested that, without eye movement, a large field of motion in the visual background is sufficient to induce an afternystagmus similar to negative OKAN. In contrast, eye tracking of a single moving target with a dark background led to afternystagmus of smooth pursuit in the subsequent dark condition, in which the eyes moved in the same direction as the previous moving target (29, 51). This behavior depends mainly upon eye movements, and not retinal slip, as the high gain of the smooth-pursuit tracking of a small moving target minimizes the retinal slip. We propose that this negative OKAN in darkness is an outcome of a sensory adaptation triggered by the retinal slip of a large moving field during a sustained period of optokinetic stimulation. Conversely, continued eye tracking by the smooth-pursuit system leads to a motor adaptation that yields an aftereffect of continuing eye movements in the same direction in darkness.

Negative OKAN has been previously documented in both animal and human subjects, albeit with evident variability in study designs and methods. However, with binocular viewing conditions we did not observe any negative OKAN in our healthy subject tested up to and after a 15-min optokinetic stimulation period with our experimental apparatus, instead, we observed a long-lasting positive afternystagmus (data not shown). We deduce that whilst sitting inside the optokinetic drum, our healthy subject tended to fixate sharp borders of the vertical stripes with the fovea, thus, the smooth pursuit overshadowed the optokinetic behavior during the drum rotation. In this situation, afternystagmus of smooth pursuit most likely masked the negative OKAN. Under monocular viewing conditions while tracking the stripes of the optokinetic drum, a representative healthy subject displayed afternystagmus with markedly reduced velocity in the same direction (Figures 4B,D). In contrast to the healthy subject, the INS patient exhibited afternystagmus in the opposite direction (Figures 3B,D).

Previous studies have reported that background movements of the whole visual field whilst the eyes remained fixed on a stationary central target led to afternystagmus in darkness with slow phase eye movements in the opposite direction of the preceding background movement (49, 50). This suggested that, without eye movement, a large field of motion in the visual background is sufficient to induce an afternystagmus similar to negative OKAN. In contrast, eye tracking of a single moving target with a dark background led to afternystagmus of smooth pursuit in the subsequent dark condition, in which the eyes moved in the same direction as the previous moving target (29, 51). This behavior depends mainly upon eye movements, and not retinal slip, as the high gain of the smooth-pursuit tracking of a small moving target minimizes the retinal slip. We propose that this negative OKAN in darkness is an outcome of a sensory adaptation triggered by the retinal slip of a large moving field during a sustained period of optokinetic stimulation. Conversely, continued eye tracking by the smooth-pursuit system leads to a motor adaptation that yields an aftereffect of continuing eye movements in the same direction in darkness.

In our pilot study with zebrafish larvae that do not possess a fovea, we recorded only a robust negative OKAN (without observing positive OKAN) in darkness after cessation of continuous optokinetic visual stimulation (unpublished data). Rats, also afoveal, exhibited both positive and negative OKAN in darkness if the preceding OKN reached a steady-state velocity (42). We interpreted the data as indicating that without foveal tracking by a smooth-pursuit system, the optokinetic system might undergo an adaptive process, most likely *via* a sensory adaptation related to the input of a continuous retinal slip signal, resulting in subsequent afternystagmus in the dark. Rabbits (37–39) and cats (40, 41), which have visual streaks, as well as monkeys (35, 36) and humans (32), who have foveas, were all reported to show afternystagmus in darkness in both directions after optokinetic stimulations. However, two components are known to contribute to the afternystagmus moving in the same direction of the preceding visual stimuli: positive OKAN and the afternystagmus of smooth pursuit. It is difficult to differentiate these two mechanisms, particularly in foveated animals. In a previous study, rabbits were reported to exhibit afternystagmus for 50 min in darkness in the same direction as the previous 15-h visual stimulation (39). However, such long-lasting afternystagmus does not match our current knowledge of positive OKAN, the duration of which is typically up to 1 min. Rather, these data suggested that the visual streak could be trained to perform smooth-pursuit tracking, an observation which was reported in a study in cats (52).

In addition to the maladaptive eye movements, INS is often linked to OA with foveal hypoplasia, a condition in which smooth-pursuit function was found to be impaired (53, 54). In contrast, in healthy humans the smooth-pursuit system dominates the optokinetic system with a much higher gain of tracking. Without a healthy smooth-pursuit system, INS patients generally present lower or even reversed (53–55) optokinetic gains during motion tracking and hence maintain considerably higher retinal slip velocities compared to healthy subjects who can rely on the smooth-pursuit system for almost perfect tracking. Following a sustained period of visual motion stimulation, the mechanisms underlying afternystagmus of smooth pursuit and negative OKAN may mask or cancel each other, depending on which of the two and/or how much of each tracking system has been activated during the visual motion stimulation.

In our present study, the INS patient showed a clear reversal of the beating direction during and after the visual stimulation, which we never recorded in our healthy subject. Since the INS patient lacks a normal smooth-pursuit function, the afternystagmus in darkness with an opposite beating direction most likely was an unmasked negative OKAN. In the healthy control, in contrast, the negative OKAN was probably masked by the afternystagmus of smooth pursuit, as the smooth-pursuit system dominated the optokinetic system during the visual stimulation. In other words, with the same experimental paradigm we would expect to record more pronounced negative OKAN in patient populations affected with nystagmus and/or foveal defects such as macular hypoplasia and age-related macular degeneration.

### Set-Point Adaptation and Ocular Motor Behavior

Negative OKAN is usually recorded in the laboratory under specific experimental conditions and not in the natural environment. However, this does not mean that the neural circuits underlying this behavior are superfluous. On the contrary, these circuits could provide an important environmental advantage. We propose that the negative OKAN is a demonstration of retinal slip velocity set-point adaptation, similar to the recently discovered vestibular set-point adaptation elicited by magneto-hydrodynamic stimulation using a MRI machine (56, 57). This adaptation is hypothesized to work as a calibration between the eye movement velocity and the retinal slip velocity, similar to earlier proposals by Leigh et al. (58). Environmental changes and nervous system development/injuries, as well as inherent variability, all affect the accuracy of velocity detection and/or eye movements. The fundamental function of this set-point adaptation in the natural environment is to provide how fast “0” is as a reference value for the retinal slip. Under experimental conditions, a sustained retinal slip input during long visual stimulation shifts the set-point to an extreme value; therefore, the eyes continue to move in the dark since the ocular motor system has an incorrect “0” setting.

Sensory/motor adaptation is essential for animals to exhibit sensory–motor coordination during various actions, as well as for sensorimotor learning. However, erroneous sensory input might also be memorized and lead to problems in behaviors. The constant moving images on the retina during pathological nystagmus would be an erroneous visual input which would exacerbate the instability of the ocular motor system.

In rabbits, negative OKAN can last for 70 h following a 48-h period of visual stimulation. Furthermore, long-term optokinetic stimulation is known to regulate transcriptions and translations in rabbit’s cerebellum (59–61). The molecular and biochemical events in these neurons are commonly linked to long-term memory formation (62). We, therefore, hypothesize that constant negative OKAN could possibly lead to a long-term ocular motor instability. In other words, the new condition can be memorized and lead to a change in ocular motor behaviors over a certain period if the stimulation is of sufficiently long duration. In INS patients, spontaneous nystagmus and the lack of normal smooth pursuit can lead to continuous retinal slip input signal and a constant high gain in the ocular motor system. We propose that at an early disease stage, nystagmus in the dark may develop due to the negative OKAN. However, after a longer period of impaired motor learning, eye movements may develop in a complex and unpredictable manner, depending upon genetic, environmental, and other factors.

Instead of the retinal slip, asymmetric optokinetic signal input may also adjust the set-point. Children with MLN or INS with a latent component exhibited reversed nystagmus in the dark (28), similar to previous reports by Dell’Osso et al. (63). Latent nystagmus is commonly associated with the nasotemporal asymmetry of the optokinetic pathways (11, 64, 65). This inherent asymmetry is normally compensated for by the top-down control of the smooth-pursuit system (66, 67). The smooth-pursuit system is mal-developed in patients with amblyopia or strabismus from an early age because of the unequal visual input from the two eyes (68, 69). Without a functional pursuit system, the nasotemporal asymmetric input from the single healthy eye can lead to latent nystagmus (66, 70). In the case of MLN, it has been proposed that the nystagmus beating direction depends on the side of the healthy eye in light and changed to the direction based on its inherent/preprogrammed dominant eye in darkness (28). Another possible explanation is that the asymmetric signal not only drives the eyes to move to the contralateral side of the healthy eye, but also adjusts the set-point of the optokinetic system.

Set-point adaptation presents in a variety of different behaviors. Similar to the optokinetic system, the vestibular system shows set-point adaptation of velocity during a sustained magnetic field stimulation (56). Moreover, in a manner which is different from velocity, set-point adaptation of position can be demonstrated as gaze-evoked nystagmus decays and rebound nystagmus (57).

In conclusion, we propose a new hypothesis that the spontaneous nystagmus in the dark can be a negative OKAN in some of the INS patients whose nystagmus symptoms in light can be linked to aberrant visual inputs and erroneous visual processing. We further suggest that patients with foveal defects may display more pronounced negative OKAN than healthy subjects due to compromised smooth-pursuit tracking. However, our hypothesis should not infer common pathological mechanisms underlying various types of nystagmus presented in all of these patient groups. In order to identify different mechanisms, understanding the correlation between genotypes and phenotypes is important. Following our hypothesis, a longitudinal study of INS from infanthood to old age would help to understand how impaired sensorimotor learning leads to new behavioral features through brain adaptations.

## Ethics Statement

The healthy subject was a 29-year-old male, who had no ocular or ocular motor abnormalities. Both subjects described herein gave their informed consent for inclusion in this report.

## Author Contributions

MH, T-FL, and DS conceived the study. T-FL, MH, CG-K, and JH performed the experiments and analyzed the data. T-FL and MH drafted the article. All authors approved the final version of the article.

## Conflict of Interest Statement

The authors declare that the research was conducted in the absence of any commercial or financial relationships that could be construed as a potential conflict of interest.
